# Molecular Detection of Antibiotic Resistance in South African Isolates of *Helicobacter pylori*

**DOI:** 10.1155/2013/259457

**Published:** 2013-04-24

**Authors:** Nicoline F. Tanih, Roland N. Ndip

**Affiliations:** ^1^Department of Biochemistry and Microbiology, Faculty of Science and Agriculture, University of Fort Hare, Private Bag X1314, Alice 5700, South Africa; ^2^Department of Microbiology and Parasitology, Faculty of Science, University of Buea, P.O. Box 63, Buea, Cameroon

## Abstract

Rapid diagnosis and treatment of *Helicobacter pylori* (*H. pylori*) presents a challenge. We aimed at investigating the presence of *H. pylori*, susceptibility profile, and associated mutations in an effort to validate the effectiveness of GenoType HelicoDR assay in *H. pylori* typing in our environment. Two hundred and fifty-four biopsy specimens were cultured and DNA extracted from seventy-eight positive cultures using the Qiagen DNA extraction kit. The GenoType Helico DR which employs reverse hybridisation was used to confirm the presence of *H. pylori*, determination of its susceptibility to antimicrobials, and detection of mutations conferring resistance to clarithromycin and fluoroquinolones. The organism was isolated from 168/254 (66.1 %) of the specimens by culture. Of the 78 strains used for further investigation, 12/78 (15.38%) were resistant to clarithromycin while 66/78 (84.61%) were susceptible. For fluoroquinolone, 70/78 (89.74%) strains were susceptible while 8 (10.26%) were resistant. Mutations were observed in 17 strains with A2147G being the most prevalent; A2146C and D91N were the least. The reverse hybridisation assay is an easy and fast technique in confirming the presence of *H. pylori*, its antimicrobial profile, and associated mutations. Analysis regarding the suitability of this assay for *H. pylori* typing is warranted in other regions.

## 1. Introduction

The burden of *Helicobacter pylori (H. pylori) *presents a tremendous challenge therapeutically [1, 2]. Clinical management of *H. pylori* infection seems tenacious because the organism lives in an environment not easily accessible to many medications, the overwhelming presence of antibiotic resistance and poor patient compliance [3]. Eradication of the organism from the stomach results in significant remission from diseases related to the pathogen [2, 4]. Regimens of choice employed for eradication currently involve the use of combination therapies: a proton pump inhibitor (PPI) or bismuth compounds and two antibiotics most commonly clarithromycin and metronidazole and/or amoxicillin [5].

Clarithromycin currently remains the available most powerful antibiotic against *H. pylori* with very low minimum inhibitory concentration compared to other molecules [6]. Fluoroquinolone such as ciprofloxacin has been incorporated in the treatment regimens after repeated treatment failures and quinolone-based triple therapies have been shown to be highly effective to patients [3, 7]. However, *H. pylori* can become resistant to these compounds, which jeopardize the success of treatments [3, 8]. Particularly, resistance to clarithromycin in *H. pylori* isolates is regarded as a main cause of treatment failure in developing countries [6]. The organism is known for its wide genetic diversity which varies geographically [9], and hence antimicrobial susceptibility profiles also vary demographically. Resistance is high in naive patients and even higher in patients suffering from unsuccessful eradication therapy [8]. Resistance to clarithromycin has been linked to decrease binding of the macrolides to the 50S bacterial ribosomal subunit [6, 10]. Extensive studies have demonstrated that point mutations in the peptidyltransferase region encoded in domain V of 23S rRNA are responsible for the organisms' resistance to clarithromycin [4]. These mutations are able to inhibit the binding between clarithromycin and the ribosomal subunit dedicated to the specific antibiotic related protein synthesis. Mutations frequently associated with clarithromycin resistance are the transitions in A2143C and A2142C positions of rRNA whilst substitution in A2142C is less frequent [6, 11, 12]. Different mutation types have been described from studies in different parts of the world amongst which are A2115G, G2141A, T2117C, T2182C, T2289C, G22-4A, C2245T, and C2611A [6]. Besides the low frequency, the clinical relevance of A2115G, G2141A, T2117C and T2289C is not well established [6, 7]. Quinolones exert their antimicrobial effects by affecting the A subunit of the DNA gyrase, the only known target enzyme in *H. pylori* [7, 13]. Resistance is associated with mutation in the gyr87 and gyr91 locus or complete absence of the wild type loci. Possible mutations found on loci 91 are D91N, D91G, D91Y, and D91A as well as N87H, N87I, N87K, or N87Y found on position 87 [3, 13, 14]. 

Resistance of *H. pylori* to antibiotics is currently widely determined in clinical bacteriology laboratories by standard methods, such as disk diffusion, microbroth dilution assay or Etest [15, 16]. These phenotypic methods are efficient in discriminating between susceptible and resistant strains, but results can be obtained only after several days (considering that *H. pylori* needs about 3–7 days to grow) and do not give insight to the type of mutation present, which could be of epidemiological and clinical significance. Conventionally, mutations are detected using molecular typing schemes such as PCR restriction fragment length polymorphism (PCR-RFLP) and sequencing [4, 11, 12, 17]. In the literature, however, there is a dearth of knowledge on mutations that occur at codons 2146-2147 for clarithromycin resistant strains [18]. A PCR-based hybridization method (Hain Life Sciences, Nehren, Germany) using a strip designed for detection of *H. pylori* and mutations at codons 2146-2147 (A2146G, A2146C, and A2147G) in 23S rRNA gene in clarithromycin resistant isolates and mutations at codon 87 and 91 (N87K, D91N, D91G and D91Y) which are associated with resistance to fluoroquinolone were employed and validated in a study by Cambau et al. [18] in France. This reverse hybridisation assay offers a one-step detection of the presence of *H. pylori, *its antimicrobial profile, and mutations associated to clarithromycin and fluoroquinolone. We sought therefore to employ the use of this assay to investigate the presence of *H. pylori* and associated mutations to these antibiotics in an effort to validate its effectiveness in *H. pylori* related studies in the environment of the Eastern Cape province of South Africa due to its high prevalence reported in our previous studies [2, 16].

## 2. Materials and Method

### 2.1. Bacterial Strains and Minimum Inhibitory Concentration

In this study, 168/254 (66.1%) of the specimens collected from patients who underwent endoscopic examination for upper gastrointestinal problems with no history of treatment with macrolide and fluoroquinolone antibiotics were found positive for culture. Seventy-eight of the positive strains isolated from 50 males and 28 females were used for further investigations. Antral and corpus gastric mucosal biopsy specimens were taken from each dyspeptic patient. The biopsies were immediately placed in sterile bijou bottles containing 0.2 g/L of cysteine and 20% glycerol in brain heart infusion (BHI) broth and transported in ice to the laboratory within 2 h of collection for culture [2]. 

Biopsies were homogenised under aseptic conditions in 0.2 g/L of cysteine and 20% glycerol in BHI broth and a loop full plated primarily on freshly prepared Columbia agar base (Oxoid, Basingstoke, England) supplemented with 7% sheep's blood (Oxoid, England) and Skirrow's supplement (Oxoid, England); trimethoprim (2.5 mg), vancomycin (5 mg), cefsulodin (2.5 mg), and amphotericin (2.5 mg) were also added to the medium. All plates were incubated at 37°C for 3–5 days under microaerophilic conditions (5%-6% O_2_, 10% CO_2_, 80%–85% N_2_) (Anaerocult, Basingstoke, England). Isolates were identified based on colony morphology and positive oxidase, urease, catalase tests, and confirmation by amplification of the *glmM* gene as previously reported [2]. Confirmed isolates were suspended in 20% glycerol and stored at −80°C in a freezer (Sanyo, Japan) until genotyping was performed. *H. pylori* reference strain NCTC 11638 was included in all experiments. Approval for this study was obtained from the Research Ethics Committee of the University of Fort Hare and the Eastern Cape Department of Health (protocol number EcDoH-Res 0002).

Minimum inhibitory concentration (MIC) was determined for clarithromycin and ciprofloxacin as previously described [16]. MIC values for the antibiotics were 0.0625–256 *μ*g/mL for ciprofloxacin and 0.125–256 *μ*g/mL for clarithromycin.

### 2.2. PCR Method

DNA was extracted from 78 strains using QIAamp tissue kit (Qiagen DNA extraction kit, SA) following the manufacturer's recommendation closely. Amplification of the bacterial DNA was done using hot-start DNA polymerase (Hain Lifescience, Nehren, Germany). Biotinylated primers were used for this study and were provided in the amplification kit. Primers were designed using the gene sequence from GenBank accession number NC_009151. Polymerase chain reaction for a single mixture had a final volume of 50 *μ*L containing 35 *μ*L primer/nucleotide mix (PNM), 5 *μ*L 10x polymerase incubation buffer, 2 *μ*L of 1.5 mM MgCl_2_, 3 *μ*L of nuclease free water (Hain Lifescience, Nehren, Germany) 0.2 *μ*L Thermo-Start Taq DNA polymerase (1-2 units were added to each tube), and 5 *μ*L DNA template. PCR was performed with a thermal cycler (Applied Biosystem, SA). The amplification cycles consisted of an initial hot start of 95°C for 15 min, initial denaturation of target DNA at 95°C for 5 min, denaturation at 95°C for 30 sec and 58°C for 2 min, primer annealing at 95°C for 25 sec, 53°C for 40 sec, and 70°C for 40 sec, and extension at 70°C for 8 min. All reactions were performed through 32 cycles (Hain Lifescience, Nehren, Germany). 

### 2.3. GenoType HelicoDR Analysis

Confirmation of isolates as *H. pylori,* antimicrobial susceptibility, and mutational analysis to clarithromycin and fluoroquinolone was performed using the GenoType HelicoDR kit (Hain Lifescience, Nehren, Germany). The kit employs the use of reverse hybridisation performed using hybridisation trays and TwinCubator (Hain Lifescience, Nehren, Germany) according to the manufacturer's instructions. Briefly, 20 *μ*L of amplified DNA was denatured and added to biotinylated probes on the strip and the hybrids formed were detected by enzyme-linked immunosorbent assay (ELISA) upon addition of substrate conjugate and substrate. Four gyr87 wild type probes (gyr87WT1–gyr87WT4) and one mutant probe (gyr87MUT), one wild type probe (gyr91WT1), and three mutant probes (gyr91MUT1–gyr91MUT3) were used for detecting fluoroquinolone resistance at position 87 and 91, respectively. For clarithromycin, one wild type probe (23SWT) and three mutant probes (23SMUT1–23SMUT3) were used for detecting resistance. On the strip, were designated conjugate control (CC), amplification control (AC) and *H. pylori* (HP). When one of the WT probes stained positive together with the gyr91WT as well as 23SWT and no mutation band formed, the results were interpreted as susceptible to the respective antibiotic. The presence of a band at CC and AC meant that the conjugate control and amplification control were in the right frame while at HP implied presence of *H. pylori* according to the manufacturer's instruction (Hain Lifescience, Nehren, Germany). 

### 2.4. Statistical Analysis

Epi Info version 2000 (Center for Disease Control and Prevention, Atlanta, GA., USA) was used for statistical analysis. Chi square or Fischer exact test was applied to test whether differences in susceptibility/resistance between values of males and females were significant at *P* value <0.05. The sensitivity and specificity of the GenoType HelicoDR kit for detection of resistance to clarithromycin and fluoroquinolone, respectively, were calculated as previously described [18]. 

## 3. Results

### 3.1. *Helicobacter pylori* Strains

In this study, 168/254 (66.1%) specimens were positive for *H. pylori *by culture and confirmed using polymerase chain reaction/reverse hybridisation assay (GenoType HelicoDR). Seventy-eight of the 168 strains were used for further investigations. 

### 3.2. Antimicrobial Susceptibility and Mutational Analysis

Seventy (89.7%) of the 78 strains were susceptible to fluoroquinolone while eight (10.3%) were resistant. For clarithromycin, 66/78 (84.6%) were susceptible and 12/78 (15.4%) were resistant. The sensitivity and specificity of detecting resistance were 98% and 100% for clarithromycin and 89% and 93% for fluoroquinolone, respectively. 

Of the 78 strains employed for further analysis, 28 (35.9%) were from females and 50 (64.1%) from males. Prevalence of clarithromycin resistance in females and males was 32.1% (9/28) and 6% (3/50), respectively, while for fluoroquinolone it was 17.9% and 6%, respectively. A higher prevalence of resistant isolates was observed in female compared with male subjects in this investigation. There was statistically significant difference with the use of clarithromycin for both sexes (*P* = 0.006), although not statistically significant for fluoroquinolone (*P* = 0.127).

Some mutations designed in line with the kit to detect resistance to clarithromycin and fluoroquinolone were delineated. Mutations observed in 17 strains are summarised in Table 1. 

Three strains had 2 or more mutations with the highest number of mutations (4) occurring in 252C. A2147G mutation associated with resistance to clarithromycin was the most prevalent mutation type in this study while A2146C and D91N were the least. Twelve of the 17 strains studied possessed A2147G mutation (Table 2). 

The frequency of A2146C mutation was very low occurring in only one (252C) of 17 strains (5.8%). The MIC of clarithromycin for these mutants (A2147G and A2146C) ranged from 32 to 256 *μ*g/mL. 

Of the 8 strains found to be resistant to fluoroquinolone, all (100%) possessed N87K mutation associated with fluoroquinolone. No designated mutation was found in five strains (247C, 253C, 369A, 249A, and 249C) using this assay as there was the complete absence of the gyrase 87 wild type indicating a mutation (designated as N87K mutation). Also, D91N associated with resistance to fluoroquinolone was detected in 1 (5.8%) of the 17 strains whilst D91Y and other designated mutation associated with fluoroquinolone resistance were not found. Minimum inhibitory concentration for fluoroquinolone (247C, 253C, 369A, 249A, and 249C) (Table 1), ranged from 8 *μ*g/mL to 32 *μ*g/mL. Strains with mutations for both clarithromycin and fluoroquinolone (252A and 252C) had MIC 32 *μ*g/mL while 247A had 256 *μ*g/mL.

## 4. Discussion

Clarithromycin and fluoroquinolone are presently the drugs of choice employed for triple combination therapy in the treatment of *H. pylori *infection [6]. Resistance to these drugs is emerging and presents a challenge. Different studies all over the world have reported resistance to clarithromycin and fluoroquinolone [8, 12, 13, 19]. The high resistance rates to these antibiotics, the burden of *H. pylori* infection, and its associated disease conditions coupled with the difficulties of rapid diagnosis and management of patients [16] necessitated the determination of the antibiogram and associated mutation to clarithromycin and fluoroquinolone to *H. pylori *strains isolated from the Eastern Cape province known to have a high prevalence of *H. pylori*-related morbidities [2, 16] using the GenoType HelicoDR assay. 

*H. pylori* was detected in 168/254 (66.1%) of the specimens studied. Clarithromycin is used worldwide as one of the potent antibiotics in the eradication therapy of *H. pylori* [4, 17]. However, resistance to clarithromycin has been increasingly reported in several studies [12, 17]. This led to the introduction of new treatments such as the fluoroquinolones which seemingly is offering great hope, but unfortunately resistance to them is emerging [7, 13]. The presence of resistance is often associated with failure of eradication therapy [20]. Findings from this study revealed moderate rate of resistance to clarithromycin and fluoroquinolone with percentages of 15.38% and 10.26%, respectively. This result corroborates the finding of Kim et al. [12] who reported resistance to clarithromycin with a range of 7.6% to 18.6% in Korea. Macrolides like clarithromycin are expensive; however, cross-resistance linked with the use of other less expensive macrolides may be responsible for this resistance. Worthy of note is the fact that clarithromycin susceptible and resistant strains have been isolated from patients with no history of exposure to macrolides [21]. Therefore, it is imperative to guide empiric treatment since administration of clarithromycin can be selected for resistance. 

The moderate rate of resistance of 10.26% to fluoroquinolone reported in this study ties with the findings of Wang et al. [13] who reported 15.6% resistance to ciprofloxacin in their study in Alberta, Canada. However, generally low resistance to the fluoroquinolones has been reported compared to other antibiotics. For example, in our previous study [16], all strains were susceptible to ciprofloxacin as opposed to the 10.26% resistance generally reported for the fluoroquinolone in the current study. We may relate this to the difference in strains as well as the methods (phenotypic versus molecular) used in both studies. Also, the strips in the GenoType HelicoDR assay are designed to generally target the fluoroquinolones. Hung et al. [3] also documented 5.7% resistance of their strains to ciprofloxacin, and Kohanteb et al. [22] reported 4.7% in their study. Isolates from Belgium, France, Italy, and Germany have higher resistance rates to ciprofloxacin or levofloxacin ranging between 16.8% and 23% [3, 23]. Also, higher resistance rates (ciprofloxacin: 33.8%; levofloxacin: 21.5%) have been observed in Japan [19]. These disparate rates of resistance could be attributed to geographical region and drug usage differences [9]. 

The prevalence of antibiotic resistance was higher in males than in females in this study reaching statistical significant difference for clarithromycin (*P* = 0.006), but not for fluoroquinolone. Importantly, more males were recruited for this study than females and this could account for the difference observed. Noteworthy is the fact that the number of strains resistant to clarithromycin (12) and fluoroquinolone (8) is almost the same, suggesting that these antibiotics could be close in their suitability as drugs of choice for *H. pylori* treatment in our environment corroborating our previous finding [16].

Mutations associated with resistance to these antibiotics were investigated. Three strains showed 2 or more mutations with the highest number of mutations occurring in 252C (Table 1). We observed that the higher the number of mutations per strain, the higher the MIC value of that strain. Strains in our study which were resistant to clarithromycin with mutation A2147G had MIC values that ranged from 32 to 256 *μ*g/mL. Strains 252A and 252C had MIC of 32 *μ*g/mL, respectively, while 247A had MIC 256 *μ*g/mL; they all possessed multiple mutations; 247C and 253C had MIC values of 8 *μ*g/mL and 16 *μ*g/mL (Table 1), respectively. These variations could be due to strain diversity. 

Fluoroquinolone acts by inhibiting DNA gyrase, topoisomerase, and interfering with bacterial DNA replication; since topoisomerase is not found in the *H. pylori* genome, mutation in the *gyrA* gene which encodes DNA gyrase is considered to be the major cause of resistance to fluoroquinolones [7]. The codons N87 and D91 are recognised as the most important target sites for ciprofloxacin binding [3, 13]. The N87H, N87I, N87K, and N87Y as well as D91G, D91N, and D91Y mutations in *gyrA* have been reported in fluoroquinolone-resistant *H. pylori *strains [3]. The assay used in this study was designed to depict N87K, D91N, D91G, and D91Y which have been frequently reported. However, N87K and D91N mutations were the only mutations associated with fluoroquinolone found in this study. N87K was the most prevalent mutation (8/17; 47.05%) associated with fluoroquinolone amongst our strains whilst D91N was found in one strain (Table 2). 

Our findings are in line with those of Wang et al. [13] and Hung et al. [3] who reported the presence of these mutations in their studies, respectively. Furthermore, it confirms the fact that these mutations are the most frequently found mutations [3, 24]. N87K was found in eight strains. The D91Y and D91G mutations were not found in the current study. This contradicts the finding of Garcia et al. [14] who reported the presence of these mutations in six and seven strains, respectively. However, occurrence in just five and two strains of their huge sample size may imply a generally low occurrence. 

Clarithromycin acts by inhibiting protein synthesis by binding to the peptidyltransferase loop of 23S rRNA which has been shown at residues A2058 and A2059 in the 23S rRNA gene of *E. coli*. When mutation occurs in these residues, the binding affinity of clarithromycin to ribosomes is reduced, resulting in clarithromycin resistance [11, 21]. The assay used in this study was designed to target the presence of A2147G, A2146G, and A2146C associated with clarithromycin resistant strains [18]; there is a dearth of information in the literature on A2147G, A2146G and A2146C mutation compared to A2142G, and A2143G which are frequently reported [6, 7] to be associated with clarithromycin resistance [12]. 

Twelve (70.5%) of the 17 strains reported to be resistant in this study had A2147G mutation (Tables 1 and 2). This accords with the findings of Cambau et al. [18] who reported a high prevalence of A2147G mutation amongst their strains. The high occurrence of A2147G mutation suggests its high frequency amongst our local strains as opposed to A2146C which occurred in one (252C) of the 17 strains studied. The sensitivity and specificity of detecting resistance to clarithromycin and fluoroquinolone observed in our current investigation corroborate with those of Cambau et al. [18], although they specifically used levofloxacin, which is also a member of the fluoroquinolone. They reported values of 94% and 99% for clarithromycin and 87% and 99% for levofloxacin, respectively. 

In conclusion, this study revealed a moderate rate of resistance to fluoroquinolone and clarithromycin, with A2147G and N87K being the main mutations associated with clarithromycin and fluoroquinolones, respectively. However, continuous surveillance of fluoroquinolone and clarithromycin resistance in *H. pylori* is thus relevant in this area to guide empiric treatment. 

## Figures and Tables

**Table 1:** Mutations associated with resistance to clarithromycin and fluoroquinolone.

Mutant strain	Number of independent mutations analysed	Mutant alleles^a^		
		Designation	Amino acid change	MIC (*µ*g/mL)
245A	1	*23SMUT3 *	A2147G	256
245C	1	*23SMUT3 *	A2147G	32
247A	2	*23SMUT3* No *gyr 87* WT	A2147G N87K	256
247C	1	No *gyr 87* WT	N87K	8
249A	1	No *gyr 87* WT	N87K	32
249C	1	No *gyr 87* WT	N87K	16
252A	2	No *gyr 87 *WT *23SMUT3 *	N87K A2147G	32
252C	4	No *gyr 87* WT *gyr 91* WT * 23SMUT2* *23SMUT3 *	N87K D91N A2146C A2147G	32
253C	1	No *gyr 87* WT	N87K	16
254A	1	*23SMUT3 *	A2147G	256
119A	1	*23SMUT3 *	A2147G	32
275A	1	*23SMUT3 *	A2147G	128
305A	1	*23SMUT3 *	A2147G	64
305C	1	*23SMUT3 *	A2147G	128
369A	1	No *gyr 87* WT	N87K	16
436C	1	*23SMUT3 *	A2147G	256
499C	1	*23SMUT3 *	A2147G	64

^a^Distinct mutation identified is given an allele name (designation), which represents a specific change at the nucleotide (nt) in a gene and a corresponding amino acid change in the gene product.

**Table 2:** Distribution of clarithromycin and fluoroquinolone mutations amongst 17 strains.

Mutations	Total number of strains	% of strains with each mutation type
A2147G	12	70.5 (12/17)
A2146C	1	5.8 (1/17)
N87K	8	47.05 (8/17)
D91N	1	5.8 (1/17)

Total number of mutation types	22	
